# Communicable and Non-communicable Diseases Diagnosis and Treatment Service Availability at Primary Health Care Units During COVID-19 Outbreak in Ethiopia

**DOI:** 10.4314/ejhs.v33i2.3S

**Published:** 2023-10

**Authors:** Tajebew Zayede Gonete, Netsanet Abera Asseffa, Kassahun Dessie Gashu, Binyam Tilahun, Dessies Abebaw Angaw, Meskerem Jisso, Alemu Tamiso, Akalewold Alemayehu, Rekiku Fikre, Biru Abdisa, Habtamu Sime, Elias Ali Yesuf, Abdurezak Umer, Mesfin Kebede, Hussen Mohammed, Bekele Yazie, Kassu Ketema Gurmu, Berhanu Fikadie Endehabtu

**Affiliations:** 1 University of Gonder, College of Medicine and Health Science, Institute of Public Health, Gonder, Ethiopia; 2 Hawassa University, College of Medicine and Health Sciences, Hawassa, Ethiopia; 3 Jimma University, Institute of Health, Jimma, Ethiopia; 4 Dire Dawa University, College of Medicine and Health Sciences, Dire Dawa, Ethiopia; 5 World Health Organization Country Office for Ethiopia, Universal Health Coverage/Life Course, Health System Strengthening Team, Addis Ababa, Ethiopia

**Keywords:** Essential health services, Communicable and non-communicable disease, primary healthcare, COVID-19, Ethiopia

## Abstract

**Background:**

Non-communicable diseases (NCDs) pose a substantial global health challenge, resulting in an annual death toll of over 15 million individuals aged 30 to 69. Ethiopia, categorized as COVID-19 vulnerable, grapples with NCD treatment challenges. This study aims to assess disease service availability at primary health units in Ethiopia during the pandemic.

**Methods:**

A facility-based cross-sectional study was conducted from October to December 2021 across regions, encompassing 452 facilities: 92 health centers, 16 primary hospitals, 344 health posts, and 43 districts. Facility selection, based on consultation with regional health bureaus, included high, medium, and low performing establishments. The study employed the WHO tool for COVID-19 capacity assessment and evaluated services for various diseases using descriptive analysis.

**Results:**

Results reveal service disruptions in the past year: hospitals (55.6%), health centers (21.7%), districts (30.2%), and health posts (17.4%). Main reasons were equipment shortages (42%), lack of skilled personnel (24%), and insufficient infection prevention supplies (18.8%). While tuberculosis treatment was fully available in 23% of health posts and malaria services in 65.7%, some health centers lacked HIV/AIDS, cardiovascular, mental health, and cervical cancer services. Most communicable and non-communicable disease diagnoses and treatments were fully accessible at primary hospitals, except for cervical cancer (56.3%) and mental health (62.5%) services.

**Conclusion:**

Significant gaps exist in expected services at primary health units. Improving disease care accessibility necessitates strengthening the supply chain, resource management, capacity building, and monitoring systems.

## Introduction

Non-communicable diseases (NCDs) are a major global public health concern because of their high morbidity and mortality rates. More than 15 million people between the ages of 30 and 69 die each year from non-communicable diseases (NCDs), with 77% of all NCD deaths occurring in low- and middle-income countries. Cardiovascular diseases, cancers, diabetes and respiratory diseases account for more than 80% of all NCDs mortality (1). Over the next 20 years, NCDs are predicted to cost the global economy $47 trillion, accounting for 75 percent of global Gross Domestic Product (GDP) (2). On September 19, 2011, the United Nations (UN) general assembly discussed a health issue for the second time in history, following HIV/AIDS, when setting a global agenda on NCDs. Furthermore, an ambitious sustainable development goal of reducing premature mortality from NCDs by one-third by 2030 has been set (3).

Worldwide, COVID-19 has claimed more than six million lives, and the case number is getting closer to half a billion (4). The world health organization advised a whole-of-government and whole-of-society approach to combat the deadly virus (5). Several measures have been taken to contain the virus, including banning public gatherings of any form, curfew, restriction of movement and flights to and from countries with high incidence among them (6, 7).

According to the World Health Organization (WHO), disruptions in service delivery for NCDs during the COVID-19 pandemic were significant. Reports indicate that 57% of countries experienced service disruptions for hypertension management, 53% for treating diabetes and its complications, and 46% for treating cancer (8, 9). During the COVID-19 pandemic in Kenya, access to non-communicable disease (NCD) medicines was disrupted at three levels: service delivery, health facility information systems, and the medicines supply chain to health facilities (9).

NCDs account for 37% of all deaths, with an estimated 275,000 deaths in Ethiopia (10). A national study emphasizing policy recommendations identified critical factors for effectively addressing the NCD burden, including political commitment, sustainable financial mechanisms, increased health workforce, availability of essential NCD drugs at primary healthcare level, and community engagement (11). However, during the COVID-19 pandemic, patients with NCDs faced challenges in seeking care due to various reasons such as fear and lack of transportation (9). Additionally, Ethiopia is categorized as one of the most vulnerable countries in terms of delivering treatment and control measures to people with both communicable and non-communicable diseases. Therefore, the aim of this study is to assess the availability of diagnosis and treatment services for communicable and NCDs at Primary Health Care Units (PHCUs) in four regions of Ethiopia.

## Materials and Methods

Non-communicable diseases (NCDs) are a major global public health concern because of their high morbidity and mortality rates. More than 15 million people between the ages of 30 and 69 die each year from non-communicable diseases (NCDs), with 77% of all NCD deaths occurring in low- and middle-income countries. Cardiovascular diseases, cancers, diabetes and respiratory diseases account for more than 80% of all NCDs mortality (1). Over the next 20 years, NCDs are predicted to cost the global economy $47 trillion, accounting for 75 percent of global Gross Domestic Product (GDP) (2). On September 19, 2011, the United Nations (UN) general assembly discussed a health issue for the second time in history, following HIV/AIDS, when setting a global agenda on NCDs. Furthermore, an ambitious sustainable development goal of reducing premature mortality from NCDs by one-third by 2030 has been set (3).

Worldwide, COVID-19 has claimed more than six million lives, and the case number is getting closer to half a billion (4). The world health organization advised a whole-of-government and whole-of-society approach to combat the deadly virus (5). Several measures have been taken to contain the virus, including banning public gatherings of any form, curfew, restriction of movement and flights to and from countries with high incidence among them (6, 7).

According to the World Health Organization (WHO), disruptions in service delivery for NCDs during the COVID-19 pandemic were significant. Reports indicate that 57% of countries experienced service disruptions for hypertension management, 53% for treating diabetes and its complications, and 46% for treating cancer (8, 9). During the COVID-19 pandemic in Kenya, access to non-communicable disease (NCD) medicines was disrupted at three levels: service delivery, health facility information systems, and the medicines supply chain to health facilities (9).

NCDs account for 37% of all deaths, with an estimated 275,000 deaths in Ethiopia (10). A national study emphasizing policy recommendations identified critical factors for effectively addressing the NCD burden, including political commitment, sustainable financial mechanisms, increased health workforce, availability of essential NCD drugs at primary healthcare level, and community engagement (11). However, during the COVID-19 pandemic, patients with NCDs faced challenges in seeking care due to various reasons such as fear and lack of transportation (9). Additionally, Ethiopia is categorized as one of the most vulnerable countries in terms of delivering treatment and control measures to people with both communicable and non-communicable diseases. Therefore, the aim of this study is to assess the availability of diagnosis and treatment services for communicable and NCDs at Primary Health Care Units (PHCUs) in four regions of Ethiopia.

**Ethics approval and consent to participate in data:** Ethical clearance was obtained from the Institutional Review Board of the University of Gondar, Jimma University, Hawassa University and Dire-Dawa University. Official permission letter was written from the Regional Health Bureau to Zonal health departments, Woreda Health offices and health facilities. Permission to conduct the study in each organization was asked through a letter. Informed consent was obtained from all study respondents and participants after adequate information about the study had been provided. The collected data were treated and kept confidentially.

## Results

**Organizational Governance and Structure:** More than half (54%) of the health centers were located in urban area, and most health posts (89%) were from rural area. Around 14(89%) of primary hospitals were governed by a governing board, and only 50(55%) of health centers were governed by a committee. Out of 92 health centers, only 42 (46%) health center provides inpatient services. Nine (10%) health centers had no isolated labor and delivery room. All health centers and primary hospitals had a 24-hour staffed dedicated emergency unit, and only seven (8%) of the health centers have an operating room. Besides, all selected primary hospitals had a 24-hour staffed dedicated emergency unit such as in-patient service, isolated labor and delivery, surgical ward and neonatal intensive care unit (NICU), but only 5 (85.7 %) of hospitals had both medical wards and operation rooms.

**Figure 1:**
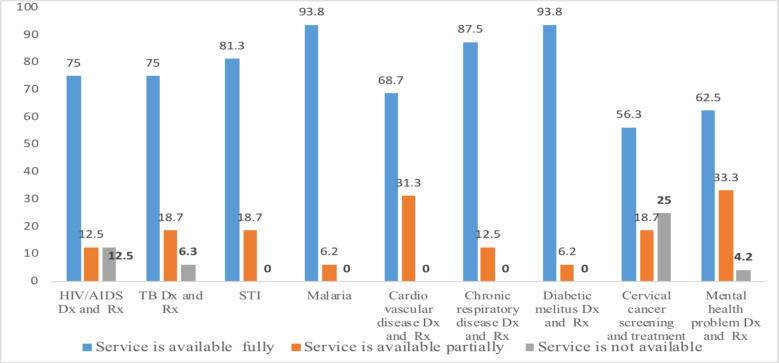
Essential health service list availability at PHCU, Ethiopia, 2021 Dx= Diagnosis, Rx = Treatment

**Essential health service list availability at PHCU and districts:** Before COVID-19 pandemic, 35 (81.4%) of woreda health offices, 67(73%) of the health center, 13(72%) hospitals, and 207 (60%) health posts had a defined list of essential health services, but after COVID-19 pandemic 30 (69.8%) woreda health offices, 58 (63%) of health centers and 229 (66.5%) of health posts received a defined list of EHS (Figure 1).

**Service interruption at PHCU and districts in the last year:** From the assessment, 13 (30.2%) of woreda health offices, 20 (21.7%) of HCs and 10 (55.6%) hospitals have reported there was a service interruption in the last year. Service interruption was the highest at primary hospitals than other PHCU.

**Common reasons for service interruption at PHCUs and districts:** Shortage of medical equipment (42%), human resources (24%), finance (15.2%) and infection prevention and patient safety supplies (18.8%) were the major reasons for service interruption at PHCUs and districts.

**Diagnosis and treatment of communicable diseases at health posts:** The assessment showed that only 93 (23%) health posts had delivered tuberculosis treatment services at full scale, and 142(41.3%) did not deliver TB treatment services. Malaria diagnosis and treatment services is available fully at 226(65.7%) of health posts, whereas 66 (19.2%) of HPs did not deliver malaria diagnosis and treatment services.

The commonest reasons for service unavailability were shortage of drugs (anti TB and malaria), medical equipment (vital sign equipment) and supplies, skill gaps, and some think it is not their scope.

**Diagnosis and treatment of communicable and NCD at health centers:** In the assessed health facilities, HIV/AIDS diagnosis and treatment (43.5%), cervical cancer screening & treatment (33.7%), cardiovascular disease (27.2%), and mental health problem diagnosis and treatment (14%) were the least available essential health services at HCs, whereas malaria (91.3%), STI (91.3), and TB (88%) diagnosis and treatment were the most available services at HCs (Table 1).

**Table 1:** Communicable and non-communicable disease prevention and control at health centers in Ethiopia, 2021. (n= 92)

Type service	Service is delivered at full scale	Service is delivered partially	Service is not available or delivered
HIV/AIDS diagnosis, prevention and control	43.5	51.1	5.4
Tuberculosis diagnosis and treatment	88	12	0
STI diagnosis and treatment	91.3	8.7	0
Malaria diagnosis and treatment	91.3	3.3	5.4
Cardiovascular disease	27.2	52.2	20.6
Chronic respiratory diseases	63	33.7	3.3
Diabetic Mellitus diagnosis and treatment	63.1	45.7	2.2
Cervical cancer screening and treatment	33.7	24	42.3
Diagnosis and treatment of mental health problems	14	24	62

STI= sexually transmitted infection.

Shortage or lack of drugs, supplies and diagnostic modalities, skill gap, absence of guidelines and protocol, shortage of trained human resource and HCs thinking it is not their scope were the common reasons for service unavailability.

**Figure 2:**
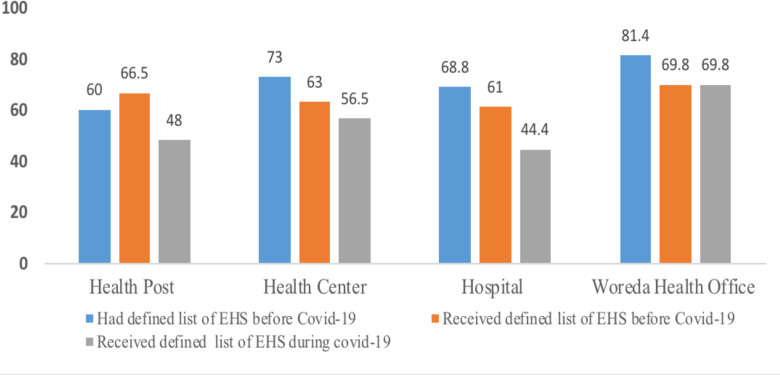
Availability of major communicable and non-communicable disease prevention and control EHS at primary hospitals in Ethiopia, 2021

**Diagnosis and treatment of communicable and non-communicable disease service availability at primary hospitals:** Most communicable and non-communicable disease diagnosis and treatment services were delivered at full scale at hospitals, whereas cervical cancer (56.3%) and mental health problems diagnosis and treatment (62.5%) services were the least available essential health services at primary hospitals compared with other essential health services (Figure 2).

Shortage of trained manpower, drugs, supplies and equipment, including diagnostics, skill gaps and some hospitals are not anti-retroviral therapy sites were among the commonest reasons for services unavailability.

**Regional variation in communicable and non-communicable disease diagnosis and treatment of service availability at primary hospitals:** Most communicable and non-communicable disease diagnosis and treatment services were available fully at primary hospitals found in Sidama and SNNP regions, whereas Amhara and Oromia regions have lower availability. Among all communicable and non-communicable disease diagnosis and treatment services, cervical cancer screening and treatment and mental health problems diagnosis and treatment had low availability (Table 2).

**Table 2:** Communicable and non-communicable disease diagnosis and treatment EHS availability at primary hospitals across regions, Ethiopia, 2021

Lists of essential health services	Oromia			Amhara			SNNP			Sidama		

	A	B	C	A	B	C	A	B	C	A	B	C
HIV/AIDS diagnosis and treatment	66.7	33.3	0	71.4	14.3	14.3	100	0	0	100	0	0
TB diagnosis and treatment	66.7	33.3	0	57.1	28.6	14.3	100	0	0	100	0	0
STI diagnosis and treatment	66.7	33.3	0	71.4	28.6	0	100	0	0	100	0	0
Malaria diagnosis and treatment	100	0	0	85.7	14.3	0	100	0	0	100	0	0
Cardiovascular disease Dx and Rx	100	0	0	28.6	71.4	0	100	0	0	100	0	0
Chronic respiratory diseases Dx & Rx	100	0	0	71.4	28.6	0	100	0	0	100	0	0
Diabetic Mellitus Dx and Rx	100	0	0	85.7	14.3	0	100	0	0	100	0	0
Cervical cancer screening and treatment	33.3	0	66.7	42.9	42.9	14.2	100	0	0	66.7	0	33.3
Mental health problems Dx and Rx	100	0	0	42.9	57.1	0	33.3	66.7	0	100	0	0

Keys: A = Service is available fully, B = Service is available partially, C = Service is not available, SNNP= South Nation, Nationalities and people, Dx= Diagnosis, Rx = Treatment

**Regional variation in communicable and non-communicable disease diagnosis and treatment service availability at health centers:** This study revealed that communicable disease diagnosis and treatment of services had low availability in the Amhara region, and HIV/AIDS diagnosis and treatment service was not available at 20%, 7.7% and 4.6 % of HCs at Dire Dawa city administration, Sidama and SNNP regions, respectively.

Except for health centers in Sidama and SNNP regions cervical cancer screening and treatment service availability was below 50%. Among regions, cervical cancer screening and treatment service was available at 62.5%, 40% and 31.8% of health centers in Oromia, SNNP region and Dire Dawa city administration, respectively. Mental health problem diagnosis and treatment service is the least available service with uniform availability across regions (Table 3). The common reasons for service unavailability were shortage of medical equipment/supplies and drugs, human resources, skill gaps, lack of laboratory diagnosis, and infrastructure.

**Table 3:** Communicable and non-communicable disease prevention and control services available at health centers across regions, Ethiopia, 2021 (n= 92)

Communicable Diseases															

Lists of essential health services	Oromia			Amhara			SNNP			Sidama			Dire Dawa		

	A	B	C	A	B	C	A	B	C	A	B	C	A	B	C
HIV/AIDS diagnosis and treatment	28	72	0	30	70	0	63.6	31.8	4.6	23.1	69.2	7.7	73.3	6.7	20
TB diagnosis and treatment	93.8	6.2	0	40	60	0	91	9		92.3	7.7	0	100	0	0
STI diagnosis and treatment	93.8	6.2	0	50	50	0	100	0	0	100	0	0	93.3	6.7	0
Malaria diagnosis and treatment	87.5	3.1	9.4	70	10	20	100	0	0	92.3	7.7	0	100	0	0
Non-communicable diseases															
Lists of essential health services	Oromia			Amhara			SNNP			Sidama			Dire Dawa		
	A	B	C	A	B	c	A	B	C	A	B	c	A	B	c
Cardiovascular disease Dx and Rx	15.6	71.9	12.5	10	60	30	18.2	54.5	27.3	15.4	38.5	46.1	86.7	13.3	0
Chronic respiratory diseases Dx & Rx	68.8	31.2	0	60	40	0	50	45.5	4.5	38.5	46.1	15.4	93.3	6.7	0
Diabetic Mellitus Dx and Rx	28	72	0	40	60	0	68.2	22.7	9.1	53.8	46.2		86.7	13.3	0
Cervical cancer screening and treatment	12.5	25	62.5	20	50	30	50	18.2	31.8	53.8	23.1	23.1	46.7	13.3	40
Mental health problems Dx and Rx	6.2	31.3	62.5	10	40	50	9.1	18.2	72.7	7.7	15.4	76.9	46.7	13.3	40

Keys: A = Service is available fully, B = Service is available partially, C = Service is not available, SNNP= South Nation, Nationalities and people.

## Discussion

According to many studies, one main reason for the attainment of Ethiopia's 2015 TB goal was increased identification and referral of individuals with possible tuberculosis cases by HEWs working in health posts (12, 13). The assessment reported that tuberculosis treatment is not available at 41.3% of health posts, whereas 12% of HC were providing partially, most citing reasons such as unavailability of drugs, reagents and supplies. The finding was supported by a national study in which interruption of laboratory reagents and supplies hindered TB-related services (14). These might lead to increased TB cases and delayed identification of cases, which is already one of the main challenges of TB service delivery and control. In addition, according to research conducted in Addis Ababa, Ethiopia's capital, on the impact of COVID19 on tuberculosis diagnosis and treatment, community health workers' TB detection decreased by 77.2 percent compared to the same time before the pandemic (15).

Moreover, more than half of assessed HCs and one-in-ten primary hospitals were providing HIV/AIDS diagnosis and treatment service either partially or not at all, which is also the main contributor to the TB-case surge. One systematic review and meta-analysis study of Ethiopia labelled it a *twin epidemic* ending its suggestion by saying intensifying case identification, INH preventive therapy and infection control are ways out, in which PHCUs play the main role (16).

Regarding malaria, diagnosis and treatment were available fully at 65.7%, 91.3% and 93.8% of assessed health posts, HCs and primary hospitals, respectively. The finding is higher than a study that only looked at HCs and hospitals at which only 36% were providing malaria diagnoses (17). The reason might relate to the time span between the two studies, more than eight years, during which many interventions might have been done in the country. Moreover, the study was only conducted in Oromia and Dire Dawa City Administration, whereas the current study included three other regions. In a review of the feasibility of malaria elimination in Ethiopia, authors reported that Ethiopia would eliminate it by 2030 or beyond if it engages more health extension workers (cadres at health posts) in malaria diagnosis and treatment (18, 19). However, more than one in three health posts were providing either partially or not all, as per our study findings.

Based on this assessment, the least available services at health centers were cervical cancer screening and mental health services, with 42.3% and 62.0%, respectively, not providing the service. Likewise, one in five and more than one-third of primary hospitals don't provide cervical cancer screening and mental health services, respectively. A review that compiled fourteen studies indicated that uptake of cervical cancer service was found to be very low at 13.4%. The same study reported that many are not even aware of the program's existence and need to improve what was recommended (20). Hence, its availability compounded with low awareness from the community might later lead to projected millions of lives lost due to cervical cancer (21). Similarly, mental health service was almost non-existent as only 14% of assessed facilities offer full service in their scope; as it has been said, it is the same here in Ethiopia *the forgotten world crisis*, most commonly cited reason relates to lack of skilled manpower and lack of clear guidelines and protocols (22).

The other group of non-communicable diseases assessed at the health center and primary hospital levels were cardiovascular, chronic respiratory and diabetic mellitus diagnosis and treatment; based on that, 72.8%, 37%, and 47.9% were provided either partially or not at all. A study assessing the cardiovascular disease burden from 1990 to 2017 reported that cardiovascular diseases are Ethiopia's primary cause of death and are considered a public health problem in Ethiopia (23). Studies have also shown significant increase in the burden of cardiovascular diseases, including hypertension in Ethiopia (24). Hence, full-service unavailability at primary health care units would be the greatest concern given its leading cause of mortality through the pooled prevalence is not more than 5% (25).

In conclusion, major communicable and non-communicable diseases diagnosis and treatment were not available as per the national standard at health posts and health centers which was reflected by partial and complete service unavailability.

TB, malaria, diabetes mellitus and chronic respiratory diseases diagnosis and treatment were the most available services both at the health center and primary hospital, while cervical cancer screening and treatment, cardiovascular disease and mental health problem diagnosis and treatment were the least available services both at health centers and hospitals, especially at health centers. There is regional variation in communicable and non-communicable disease diagnosis and treatment across regions, and SNNP and Sidama regions have higher service availability than others.

Shortage of drugs, medical equipment, supplies, diagnostic modalities, skill gap, and low rate of monitoring and evaluation were the common reasons for service unavailability across all regions. To fully avail diagnosis and treatment of communicable and non-communicable diseases at primary health care units, it is recommended to strengthen the supply chain and resource management while simultaneously enhancing capacity building and monitoring systems to ensure the availability of drugs, medical equipment, and skilled healthcare professionals.

Designing and conducting need-based capacity building, cluster-based mentorship, strengthening monitoring and evaluation, identifying, forecasting and availing essential medicines, supplies, and diagnostic modalities will improve communicable and non-communicable disease diagnosis and treatment service availability.
